# NR1D1‐Mediated Suppression of JAK1/STAT3 Signaling Contributes to the Therapeutic Effects of Triptolide in Rheumatoid Arthritis

**DOI:** 10.1155/mi/3971886

**Published:** 2026-08-25

**Authors:** Conglin Ren, Mingshuang Li, Caijian He, Kuanglin Li, Huanhong Yang

**Affiliations:** ^1^ Taizhou Hospital, Shanghai University of Traditional Chinese Medicine, Taizhou, China, shutcm.edu.cn; ^2^ Taizhou Traditional Chinese Medicine Hospital, Zhejiang Chinese Medical University, Taizhou, China, zcmu.edu.cn

**Keywords:** JAK1/STAT3 signaling, network pharmacology, NR1D1, rheumatoid arthritis, triptolide

## Abstract

**Background:**

Triptolide demonstrates potent antiarthritic effects in rheumatoid arthritis (RA), although its complete mechanism is not fully elucidated. This study aimed to systematically identify its core targets and regulatory pathways by integrating network pharmacology, molecular docking, and experimental validation.

**Methods:**

Potential targets of triptolide and RA‐associated genes were sourced from public databases. Network module analysis identified key targets, the binding affinity of which to triptolide was subsequently assessed by molecular docking, molecular dynamics (MD) simulations, and drug affinity responsive target stability (DARTS) assay. The functional effects of triptolide on RA fibroblast‐like synoviocytes (RA‐FLS) behavior, joint inflammation, and bone erosion were further investigated using in vitro cellular assays and a collagen‐induced arthritis (CIA) mouse model.

**Results:**

Our analysis identified 73 candidate targets for triptolide against RA, which were primarily enriched in biological processes such as transcription factor activity and inflammatory response. Protein–protein interaction (PPI) network analysis highlighted a core module of five genes, among which STAT3 and its upstream regulator NR1D1 were selected for subsequent investigation. Molecular docking, MD simulation, and DARTS assay confirmed the high‐affinity and direct binding of triptolide to NR1D1. In vitro, triptolide effectively suppressed RA‐FLS migration and invasion, upregulated NR1D1, and inhibited JAK1/STAT3 signaling. In vivo, triptolide administration ameliorated arthritis severity and reduced bone erosion in CIA mice, as well as modulation of the NR1D1/STAT3 axis.

**Conclusion:**

This study demonstrates that triptolide alleviates RA through NR1D1‐mediated inhibition of JAK1/STAT3 signaling. Our findings elucidate a novel molecular mechanism for triptolide’s antiarthritic effects, thereby revealing the NR1D1/JAK1/STAT3 axis as a potential therapeutic target for RA.

## 1. Introduction

Rheumatoid arthritis (RA) is an autoimmune disorder characterized by recurrent synovitis, angio‐arthritis formation, and progressive osteoarticular erosion, ultimately leading to joint deformity and even lifelong disability [1, 2]. Global epidemiologic data showed that the prevalence of RA has continued to climb over the past three decades and become a major public health burden [3]. Despite the complexity of the pathogenesis, aberrant immune activation in synovial tissue (such as T/B cell infiltration and proinflammatory factor storms) is believed to be one of the central mechanisms driving the pathologic process [4].

Early intervention is crucial for managing RA. The current treatment paradigm relies on a tiered approach, with conventional synthetic disease‐modifying antirheumatic drugs (DMARDs) like methotrexate as the first‐line cornerstone due to their proven efficacy and cost‐effectiveness [5]. For inadequate responders, biological DMARDs offer enhanced targeting and superior control of disease activity and progression [6]. Nevertheless, challenges persist, including the high cost and predominantly parenteral administration of biologics, which limit their accessibility [7]. Furthermore, all current therapies carry potential safety risks requiring monitoring, and a proportion of patients remain treatment‐resistant [8]. Therefore, investigating alternative agents, such as the natural compound, remains imperative to address these unmet needs and explore novel mechanistic pathways in RA therapy.

Triptolide has attracted much attention because of its excellent therapeutic efficacy in immune‐related and oncological diseases [9]. Zhang et al. [10] found that triptolide alleviates lupus symptoms by upregulating miR‐146a expression, thereby suppressing the TLR7/NF‐κB signaling and reducing autoantibody production. Wang et al. [11] indicated that triptolide inhibits thyroid cancer cell growth and induces apoptosis by upregulating CDKN1A/p53 and suppressing c‐JUN/NF‐κB signaling. As for RA, evidence from animal studies suggested that triptolide monotherapy or combination regimens have comparable or superior efficacy and lower rates of adverse effects than conventional antirheumatic drugs, profoundly reducing joint swelling and delaying bone erosion [12, 13]. Despite the growing number of reports on the application of triptolide, the drug’s targets of action and key regulatory mechanisms remain to be elucidated.

NR1D1, a core circadian clock component, has emerged as a critical regulator of immune and inflammatory responses, making it an attractive therapeutic target for autoimmune diseases [14, 15]. However, whether NR1D1 is involved in triptolide’s therapeutic effects in RA has not been investigated. Network pharmacology offers a systematic framework for elucidating the mechanisms of traditional drugs by constructing interactive drug‐target disease networks [16]. In this study, we employed this approach to identify the potential antirheumatic targets of triptolide and subsequently validated its efficacy in suppressing synovial inflammation and bone erosion through multilevel experiments.

## 2. Materials and Methods

### 2.1. Potential Targets of Triptolide and Causative Genes of RA

**Figure 1:**
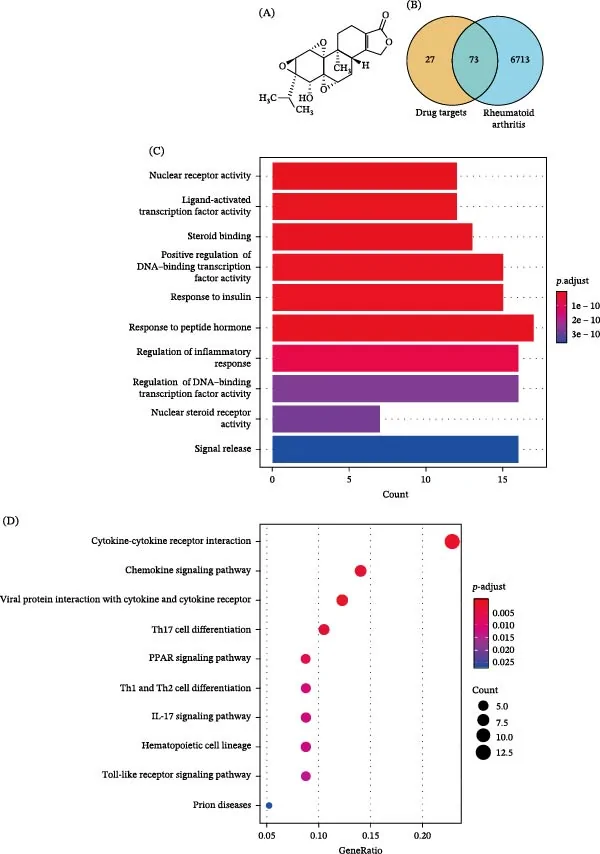
Potential targets of triptolide against rheumatoid arthritis (RA) and functional enrichment analysis. (A) Chemical structure of triptolide. (B) Venn diagram visualizing the overlap between drug targets and RA‐related genes. (C) Gene Ontology (GO) analysis for the potential targets. (D) KEGG pathway annotations for the potential targets.

First, we used the keyword “rheumatoid arthritis” to obtain potential genes associated with RA development from the GeneCards database [17]. We then queried the PubChem database for the 2D structure (Figure 1A) and SMILES string of triptolide, which were entered into the Swiss Target Prediction database for drug target prediction. Common genes in both databases were screened as candidate targets for triptolide in the treatment of RA.

### 2.2. Functional Enrichment and Interactive Analysis of Candidate Genes

Functional enrichment of candidate target genes was analyzed using the “clusterProfiler” [18] and “ggplot2” [19] packages in R software. The top 10 results of enrichment analysis were presented as bar or bubble plots (ranked by adjusted *p*‐value). For protein–protein interaction (PPI), we constructed a preliminary interactive model through the STRING database [20, 21]. Thereafter, the PPI information was transferred to Cytoscape software for in‐depth analysis and personalized presentation. In order to highlight the central module in the network, we calculated the contribution of each node, and the top five genes (ranked by degree) were filtered for subsequent studies.

### 2.3. Probing NR1D1 Expression in Public Dataset and Molecular Docking

To understand the expression pattern of NR1D1 in the RA condition, we selected the publicly available GSE55235 dataset (https://www.ncbi.nlm.nih.gov/geo/query/acc.cgi?acc=GSE55235) for further analysis, which examined knee synovial tissue from 10 RA patients and 10 healthy controls (HCs). The normalization and processing of raw data have been described in detail in our previous article [22]. For molecular docking, the SDF files of triptolide and PDB files of target genes, downloaded from the RCSB‐PDB database, were prepared. CB‐Dock2 is a computational tool for predicting the binding sites and conformations of small molecules to proteins [23]. In this scoring system, lower (more negative) Vina scores indicate higher binding affinity, with scores below −5.0 kcal/mol generally considered indicative of favorable binding.

### 2.4. Molecular Dynamics (MD) Simulation

MD simulations were performed using YASARA (version 10.3.16, Bio‐Prodict BV). The initial complex structure was first preprocessed within YASARA, which involved removing crystallographic water molecules, adding missing hydrogen atoms, and assigning protonation states at pH 7.4 to optimize the system. The simulation was conducted using the AMBER14 force field. The solvated complex was placed in a cubic simulation box with periodic boundary conditions, filled with the TIP3P water model. The system energy was first minimized using a combination of steepest descent and simulated annealing minimization to remove any bad contacts. Finally, a production MD simulation was run for 50 ns under constant temperature and pressure. The trajectory was saved every 100 ps for subsequent analysis of root‐mean‐square deviation (RMSD) and other relevant parameters.

### 2.5. Reagents

Triptolide, RIPA lysis buffer, BCA protein quantification kit, and CCK‐8 were purchased from Meilunbio Technology (Dalian, China). Protease was purchased from Sigma‐Aldrich (Saint Louis, USA). RNA extraction kit was purchased from YiShan Biotechnology (Shanghai, China). Fetal bovine serum (FBS), streptomycin/penicillin antibiotics, and DMEM medium were purchased from Gibco (New York, USA). TNF‐α recombinant protein and primary antibodies including NR1D1, JAK1, STAT3, p‐STAT3, and GAPDH were purchased from Proteintech (Chicago, USA). Primary antibody p‐JAK1 was purchased from Abcam (Cambridge, UK). ELISA kits were purchased from BOSTER Biotechnology (IL‐1β and IL‐6 kits).

### 2.6. Cell Culture and Intervention

RA fibroblast‐like synoviocytes (RA‐FLS) were purchased from Meisen Cell Technology (Jinhua, China). The culture system consisted of DMEM containing 10% FBS and 1% antibiotics. For in vitro experiments, cells were pretreated with triptolide for 2 h, followed by stimulation with 10 ng/mL TNF‑α for 24 h. Cells were divided into four groups: (1) control (untreated), (2) model (TNF‑α only), (3) TP‑L (TNF‐α + 20 nM triptolide), and (4) TP‑H (TNF‐α + 40 nM triptolide). To minimize freeze‐thaw cycles, triptolide and TNF‐α were dissolved in DMSO and distilled water, respectively, as storage solutions and then diluted to the indicated concentrations for respective studies.

### 2.7. Cytotoxicity Assessment of Triptolide

To assess the cytotoxicity of triptolide, the CCK‐8 assay was performed. RA‐FLS seeded in 96‐well plates were treated with gradient concentrations of triptolide (5, 10, 20, 40, 80, and 100 nM) for 24, 48, and 72 h, followed by incubation with CCK‐8 reagent for 2 h. Absorbance at 450 nm was measured using a PerkinElmer microplate reader (Waltham, MA, USA).

### 2.8. Drug Affinity Responsive Target Stability (DARTS) Assay

Total protein extracts from RA‐FLS were diluted with TNC buffer, prepared as described previously [24], and then treated with either triptolide (10 μM) or DMSO. After 1 h of incubation at room temperature, protease (5, 10 μg/mL) was added, and the mixture was maintained at 37°C for 20 min. The reaction was stopped with SDS‑PAGE loading buffer, and NR1D1 protein levels were subsequently detected via Western blot.

### 2.9. Wound Healing and Transwell Assays

To examine the effect of triptolide on the migratory capacity of RA‐FLS, wound healing assay was performed. RA‐FLS were seeded into six‐well plates, and after the cells were completely fused, sterile pipette tips were used to make uniform scratches; detached cells were rinsed gently with PBS three times. Afterwards, cells in serum‐free medium were pretreated with low‐dose (TP‐L, 20 nM) and high‐dose triptolide (TP‐H, 40 nM) for 2 h and then stimulated with TNF‐α (10 ng/mL) for 24 h. Images of cell migration were captured at time 0 and 24 h by an optical microscope (Nikon, Japan).

The invasive ability of RA‐FLS was detected by the Transwell assay. The upper chambers were precoated with Matrigel and incubated for 1 h. Subsequently, RA‐FLS (2 × 10^4^) in serum‐free medium were pretreated with TP‐L and TP‐H and then stimulated with TNF‐α. After 24 h of complete medium induction, the invaded cells were fixed and stained. The degree of invasion was quantitatively evaluated by the number of cells crossing the Matrigel layer.

### 2.10. RT‐qPCR

Total RNA of RA‐FLS was extracted using the RNA rapid extraction kit. After mRNA quality control, cDNA was reverse synthesized and applied to PCR amplification on the ABI‐7500 platform (Applied Biosystems, USA). Primer sequences for target genes and reference (GAPDH) are listed in Table 1. Gene expression was compared according to the 2^−△△CT^ algorithm.

**Table 1:** RT‐qPCR primers used in this study.

Gene symbol	Sequence (5′ to 3′)
NR1D1	Forward: TGGACTCCAACAACAACACAG Reverse: GATGGTGGGAAGTAGGTGGG
JAK1	Forward: GAGACAGGTCTCCCACAAACAC Reverse: GTGGTAAGGACATCGCTTTTCCG
STAT3	Forward: CTTTGAGACCGAGGTGTATCACC Reverse: GGTCAGCATGTTGTACCACAGG
GAPDH	Forward: TGTGGGCATCAATGGATTTGG Reverse: ACACCATGTATTCCGGGTCAAT

### 2.11. Western Blot

Total protein was harvested from RA‐FLS and ankle tissues of mice using RIPA lysis buffer. Upon completion of the protein quantification, equal amounts of protein lysates were separated by electrophoresis and then transferred to PVDF membranes. Afterwards, the membranes were subjected to membrane blocking and primary/secondary antibody incubation, as described previously [25]. The expression of target genes was detected using Bio‐Rad protein imaging system (California, USA) and quantitatively analyzed using ImageJ software based on band densitometry.

### 2.12. NR1D1 Silencing (siRNA) Experiments

To investigate whether NR1D1 directly regulates the JAK1/STAT3 pathway, gene‐silencing experiments were conducted. RA‐FLS and siRNA expression vectors (GenePharma, Shanghai, China) were used to establish NR1D1‐silencing cell models. The siRNA transfection procedure referred to the manufacturer’s instructions and previous research [26]. Cells were incubated for 48 h to allow mRNA degradation and protein depletion, followed by Western blot analysis to assess gene knockout efficiency.

### 2.13. Animal Modeling and Treatment With Triptolide

DBA/1J mice were obtained from GemPharmatech Co., Ltd. (Jiangsu, China). All animal experiments were approved by the Institutional Animal Care and Use Committee of Shanghai Rat & Mouse Biotechnology Co., Ltd. (Number LL‐2024‐112) and were conducted in accordance with the Guidelines for the Care and Use of Laboratory Animals (GB/T 35892‐2018). The collagen‐induced arthritis (CIA) model was induced as previously described [27, 28]. Briefly, mice were immunized intradermally at the base of the tail with 100 μL of bovine type II collagen emulsified in Freund’s complete adjuvant. A booster immunization was administered 3 weeks later. Mice were randomly divided into four groups (*n* = 8 per group) receiving daily intraperitoneal injections for 28 days as follows: (1) control (saline), (2) CIA model (saline), (3) TP‐L (CIA + triptolide 20 μg/kg), and (4) TP‐H (CIA + triptolide 40 μg/kg). Upon completion of the treatment period, all mice were euthanized by CO_2_ inhalation, and blood and joint tissues were collected for analysis.

### 2.14. Assessment of Arthritis in Mice

Arthritis in mice was assessed using a qualitative score system (0–4 points), which has been widely applied in previous studies [29, 30]. In addition, we measured the thickness of mice hind paw (every 3 days) using vernier calipers to assess the degree of edema.

### 2.15. Histopathological Assessment

The joints, livers, and kidneys of the mice were harvested immediately after euthanasia. For joint tissues, samples were fixed with 4% paraformaldehyde solution for 24 h and then decalcified for 3 weeks. Paraffin‐embedded specimens were cut into 4 µm thick slices and then subjected to H&E or Safranin O‐fast green staining. Histopathological changes were observed under an optical microscope.

### 2.16. Detection of Inflammatory Cytokines and Serum Biochemical Markers

To analyze the anti‐inflammatory effect of triptolide and its impact on hepatic toxicity, levels of inflammatory cytokines (IL‐1β and IL‐6) in culture supernatants or serum taken from mice were determined using commercial ELISA kits; levels of alanine transaminase (ALT) and aspartate aminotransferase (AST) were measured using an automated biochemical analyzer (Beckman, USA).

### 2.17. Statistical Analysis

Statistical analyses were performed with R software or GraphPad Prism 9 software (CA, USA). Continuous variables were presented as mean ± SD. Differences among groups (*N* ≥ 3) were analyzed by one‐way ANOVA. Multiple comparisons were corrected by Tukey test. It was considered statistically significant at *p* < 0.05.

## 3. Results

### 3.1. Predicting RA‐Related Genes and Drug Target Genes

First, we obtained 6786 potential RA‐associated genes from the GeneCards database using “rheumatoid arthritis” as the search term (Supporting information 1: Table S1). Simultaneously, 100 predicted target genes of triptolide were retrieved from the Swiss Target Prediction database (Supporting information 2: Table S2). The intersection of these two datasets, visualized in a Venn diagram (Figure 1B), yielded 73 overlapping genes. These core overlapping targets suggest a potential molecular basis for triptolide’s anti‐RA effects.

### 3.2. Functional Enrichment Analysis of Candidate Genes

To elucidate the biological functions of these 73 candidate genes, we performed a functional enrichment analysis. The Gene Ontology (GO) analysis (Figure 1C) highlighted significant enrichment in nuclear receptor activity and the regulation of inflammatory response, implicating these processes as key mechanisms. Furthermore, KEGG pathway analysis (Figure 1D) revealed that these genes are predominantly involved in cytokine‐cytokine receptor interaction and chemokine signaling pathway. These findings strongly suggest that triptolide may ameliorate RA by modulating immune inflammation, providing a systematic and mechanistic perspective for its therapeutic action.

### 3.3. Identify the Central Module of the PPI Network

To further explore the associations among the 73 candidate genes, we performed PPI interaction analysis with the STRING database and optimized the network, comprising 57 nodes and 128 edges, in the Cytoscape software (Supporting information 3: Figure S1). A central module consisting of five target genes (STAT3, IL1B, JUN, ESR1, and PTGS2) was calculated based on the contribution of nodes (Table 2). Considering that aberrant activation of the STAT3 pathway plays a central role in the expansion and maintenance of joint inflammation [31], we next sought to probe the role of triptolide in regulating STAT3 signaling and its upstream regulators.

**Table 2:** The attributes of the top five core genes.

Gene name	Degree	Betweenness	Closeness
STAT3	19	1203.0426	0.2062937
IL1B	14	404.95935	0.19281046
JUN	13	427.60925	0.19732441
ESR1	11	232.244	0.18910256
PTGS2	11	638.1861	0.19032258

### 3.4. NR1D1 Is a Potential Upstream Regulator of STAT3 and Molecular Docking Validation

**Figure 2:**
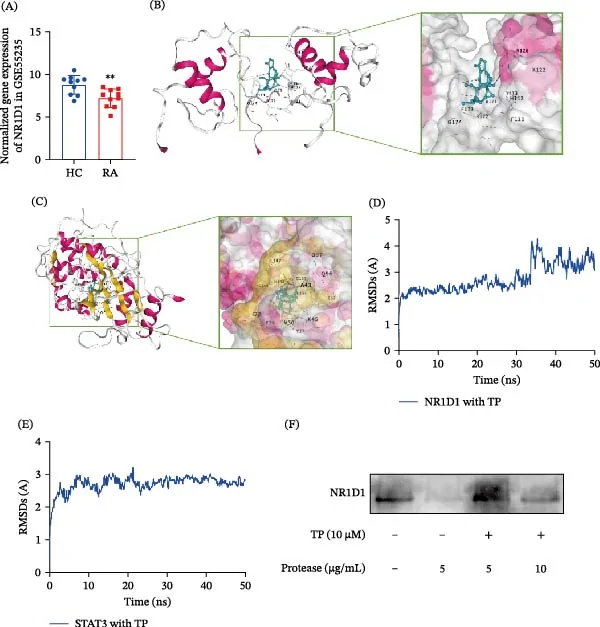
Molecular docking, molecular dynamics simulation, and drug affinity responsive target stability (DARTS) assay. (A) Normalized gene expression of NR1D1 in the GSE55235 dataset. (B) Molecular docking of NR1D1 with triptolide. (C) Molecular docking of STAT3 with triptolide. (D) Temporal variation in RMSD values for NR1D1–triptolide complex. (E) Temporal variation in RMSD values for STAT3–triptolide complex. (F) Triptolide treatment significantly protected NR1D1 from protease digestion, as determined by the DARTS assay. Data are shown as mean ± SD.  ^∗∗^ *p* < 0.01, compared with the healthy control (HC) group.

NR1D1 is a ligand‐regulated nuclear receptor and transcriptional repressor that plays a critical role in regulating the circadian rhythm and metabolism [32]. Recent studies have shown that activation of NR1D1 ameliorates multiple autoimmune disease by inhibiting the NLRP3 axis [33], microglia activation [34], and Th17 cell development [35]. To evaluate the role of NR1D1 in RA progression, we analyzed a public available dataset (GSE55235), which examined 10 RA and 10 HC synovial tissues. As illustrated in Figure 2A, the expression of NR1D1 was significantly decreased in the RA group compared with controls (normalized gene expression, 7.215 ± 1.075 vs. 8.797 ± 1.127) (*p* < 0.01). To explore whether there are interactions between triptolide and target genes (NR1D1 and STAT3), we performed molecular docking analysis. The results confirmed that triptolide exhibits robust binding affinity with these targets, with Vina scores ranging from −7.2 to −8.1 kcal/mol (Table 3). The detailed binding sites of triptolide to the targets are shown schematically in Figure 2B,C.

**Table 3:** Molecular docking results of target genes with triptolide.

Target name	Vina scores (kcal/mol)	Cavity size	Center^a^ (*x*, *y*, *z*)	Docking size^b^ (*x*, *y*, *z*)
NR1D1	−7.2	4367	5, −1, 4	20, 33, 34
STAT3	−8.1	761	16, −6, −16	20, 20, 20

^a^Docking pocket center coordinates.

^b^The size in the *x*, *y*, and *z* directions of the docking pocket.

### 3.5. MD Simulations and DARTS Assay

To further validate the stability of the key complexes identified through molecular docking, we conducted MD simulations. The RMSD of the protein backbones was analyzed to assess conformational stability. Both complexes demonstrated excellent stability, with their RMSD trajectories rapidly converging and maintaining stable plateaus with minimal fluctuations throughout the simulation (Figure 2D,E). Given that NR1D1 is the primary upstream target of interest in our study, we next performed a DARTS assay to provide direct biochemical evidence for the physical binding between triptolide and NR1D1. As shown in Figure 2F, triptolide treatment significantly protected NR1D1 from protease digestion compared with the control, indicating that triptolide directly binds to and stabilizes the NR1D1 protein.

### 3.6. Triptolide Inhibits RA‐FLS Proliferation

The cytotoxic effects of triptolide on RA‐FLS were examined by the CCK‐8 assay. Cells were exposed to various concentration gradients (5, 10, 20, 40, 80, and 100 nM) in serum‐free medium for 24, 48, and 72 h. The results showed that low‐concentration interventions did not significantly alter cell viability, while concentrations greater than 40 nM markedly inhibited the proliferation of RA‐FLS in a gradient‐dependent manner (Supporting information 3: Figure S2). According to the preliminary findings, we chose two concentrations of triptolide (20 and 40 nM) for subsequent experiments.

### 3.7. Triptolide Impedes Migration and Invasion of RA‐FLS

**Figure 3:**
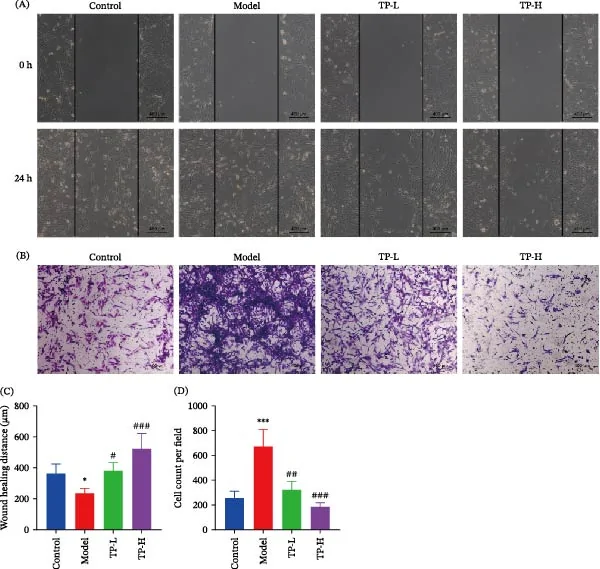
Effects of triptolide on migration and invasion of RA fibroblast‐like synoviocytes (RA‐FLS). Cells were divided into four groups: control, model (TNF‐α 10 ng/mL); TP‑L (TNF‑α + 20 nM triptolide); and TP‑H (TNF‑α + 40 nM triptolide). (A) Representative images of RA‐FLS in wound healing assay. Original magnification, ×100. (B) Representative images of RA‐FLS in Transwell assay. Original magnification, ×100. (C) Migration capacity of each group assessed by wound healing distance (*n* = 3). (D) Invasion capacity of each group assessed by cell counting (*n* = 3). Data are shown as mean ± SD.  ^∗^ *p* < 0.05 and  ^∗∗∗^ *p* < 0.001, compared with the control group. ^#^ *p* < 0.05, ^##^ *p* < 0.01, and ^###^ *p* < 0.001, compared with the model group.

The migratory and invasive properties of synoviocytes trigger joint inflammation, synovial hyperplasia, and cartilage erosion. Wound healing assay was applied to detect the migration distance of RA‐FLS under triptolide intervention. Compared with the TNF‐α–stimulated group, the migration distance of cells was significantly decreased after 24 h of triptolide (20 and 40 nM) treatment (Figure 3A,C). Meanwhile, invasive assessment showed that the number of cells passing through the Matrigel dramatically increased under TNF‐α stimulation, while triptolide hindered this invasiveness in a dose‐dependent manner (Figure 3B,D).

### 3.8. Triptolide Suppresses the Secretion of Proinflammatory Cytokines by RA‐FLS

**Figure 4:**
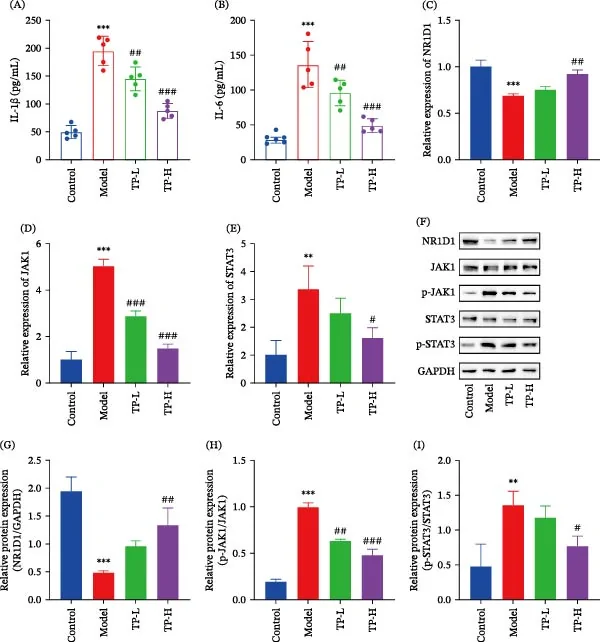
Triptolide suppressed the production of inflammatory cytokines and JAK1/STAT3 signaling through the activation of NR1D1 in RA‐FLS. Cells were divided into four groups: control, model (TNF‐α 10 ng/mL), TP‑L (TNF‑α + 20 nM triptolide), and TP‑H (TNF‑α + 40 nM triptolide). (A, B) Levels of inflammatory cytokines IL‐1β (A) and IL‐6 (B) in RA‐FLS supernatants were assessed by ELISA (*n* = 5). (C–E) Alterations in the relative expression of NR1D1 (C), JAK1 (D), and STAT3 (E) in RA‐FLS assessed by qPCR (*n* = 3). (F–I) Western blot detected changes in the relative expression of NR1D1 and phosphorylation of JAK1 and STAT3 in RA‐FLS (*n* = 3). Data are shown as mean ± SD.  ^∗∗^ *p* < 0.01 and  ^∗∗∗^ *p* < 0.001, compared with the control group. ^#^ *p* < 0.05, ^##^ *p* < 0.01, and ^###^ *p* < 0.001, compared with the model group.

For in vitro experiments, local inflammatory microenvironment was established using 10 ng/mL TNF‐α stimulation. We examined the effects of triptolide (20 and 40 nM) on the expression of proinflammatory cytokines in TNF‐α–induced RA‐FLS. Our findings suggested that both TP‐L and TP‐H effectively reduce IL‐1β (Figure 4A) and IL‐6 (Figure 4B) secretion compared to the inflammation model group.

### 3.9. Triptolide Activates NR1D1 to Inhibit JAK1/STAT3 Signaling in RA‐FLS

The JAK/STAT pathway serves as a pivotal hub in inflammatory cytokine signaling, and its excessive activation drives synovial inflammation and joint destruction [36]. Based on the above bioinformatics predictions, we assessed the expression levels of NR1D1 and JAK1/STAT3 signaling in RA‐FLS across different groups. After receiving TNF‐α stimulation, PCR results showed that NR1D1 expression was suppressed, while JAK1/STAT3 signaling was activated (Figure 4C–E). In the observation group, triptolide dose‐dependently rescued NR1D1 expression and downregulated JAK1/STAT3 signaling. Further, western blot also revealed a substantial reduction in the phosphorylation levels of JAK1 and STAT3 in triptolide‐treated groups, implying that triptolide may exert its anti‐inflammatory effect through the activation of NR1D1, which in turn inhibits the downstream JAK1/STAT3 pathway (Figure 4F–I).

**Figure 5:**
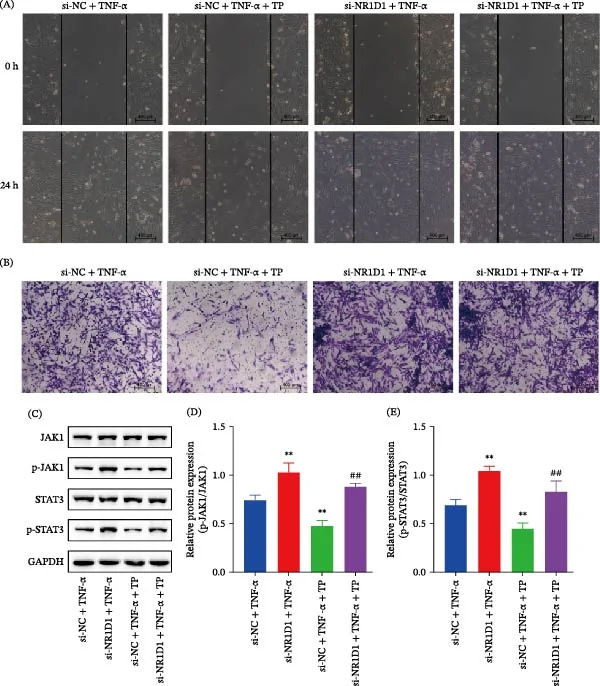
The effects of NR1D1 knockout on RA‐FLS migration, invasion, and JAK1/STAT3 signaling. RA‐FLS was transfected with siRNAs for NR1D1 (si‐NR1D1) or control siRNA (si‐NC). Cells were divided into four groups: si‐NC + TNF‐α (10 ng/mL), si‐NC + TNF‐α + TP (triptolide 40 nM), si‐NR1D1 + TNF‐α (10 ng/mL), and si‐NR1D1 + TNF‐α + TP (triptolide 40 nM). (A) Representative images of RA‐FLS in wound healing assay. Original magnification, ×100. (B) Representative images of RA‐FLS in Transwell assay. Original magnification, ×100. (C–E) Western blot detected changes in JAK1 and STAT3 phosphorylation levels in RA‐FLS (*n* = 3). Data are shown as mean ± SD.  ^∗∗^ *p* < 0.01, compared with the si‐NC + TNF‐α group. ^##^ *p* < 0.01 and ^###^ *p* < 0.001, compared with the si‐NC + TNF‐α + TP group.

To further elucidate the effects of NR1D1 inhibition on the JAK1/STAT3 signaling pathway and the anti‐inflammatory mechanism of triptolide, we established a cell model with NR1D1 silencing using siRNA technology. Western blot analysis confirmed a significant reduction in NR1D1 protein expression (Supporting information 3: Figure S3). We determined that knockdown of NR1D1 significantly enhanced the migratory and invasive capabilities of RA‐FLS. In this context, triptolide treatment failed to suppress the promoted cell migration and invasion (Figure 5A,B). Additionally, elevated levels of JAK1 and STAT3 protein phosphorylation indicate that inhibiting NR1D1 expression exacerbates the progression of inflammatory states (Figure 5C–E). Furthermore, the application of triptolide did not reverse these effects compared to the siRNA group. These findings indicate that triptolide primarily exerts its anti‐inflammatory effects through NR1D1‐mediated inhibition of JAK1/STAT3 signaling.

### 3.10. Triptolide Ameliorates Joint Inflammation and Bone Destruction in CIA Mice

**Figure 6:**
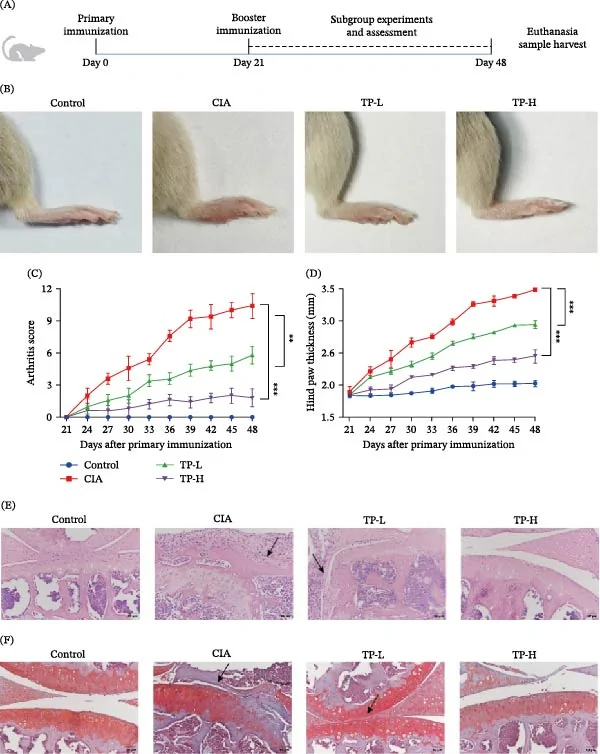
Triptolide ameliorates joint inflammation and bone destruction in collagen‐induced arthritis (CIA) mice. Mice were randomly divided into four groups (*n* = 8 per group): control (saline), CIA model (saline), TP‐L (CIA + triptolide 20 μg/kg), and TP‐H (CIA + triptolide 40 μg/kg). (A) Schematic diagram of CIA modeling and intervention approach. (B) Representative images of ankle joint from each group. (C, D) Comparison of arthritis score and paw swelling among groups of mice. (E) Representative images of H&E staining of joint tissues from each group. Original magnification, ×200. The arrow refers to synovial hyperplasia and inflammatory cell infiltration. (F) Representative Safranin O‐fast green–stained joint sections from each group. Original magnification, ×200. The arrow refers to eroded cartilage surfaces. Data are shown as mean ± SD.  ^∗∗^ *p* < 0.01 and  ^∗∗∗^ *p* < 0.001, compared with the CIA group.

**Figure 7:**
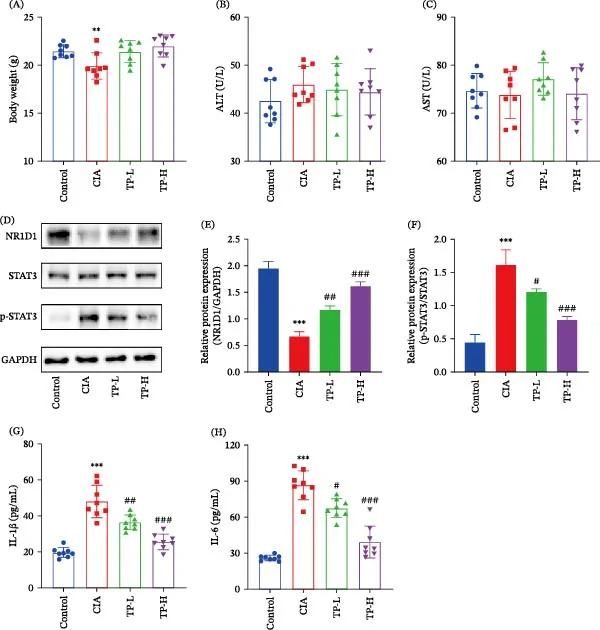
Triptolide exerts antiarthritic effects through NR1D1‐mediated STAT3 signaling inhibition in CIA mice. Mice were randomly divided into four groups (*n* = 8 per group): control (saline), CIA model (saline), TP‐L (triptolide 20 μg/kg), and TP‐H (triptolide 40 μg/kg). (A) Comparison of body weight changes of mice in each group. (B, C) Serum biochemical analysis of hepatic function markers ALT (B) and AST (C). (D–F) Western blot detected changes in the relative expression of NR1D1 and phosphorylation of STAT3 in ankle tissues of CIA mice. (G, H) Levels of inflammatory cytokines IL‐1β (G) and IL‐6 (H) in mouse serum were assessed by ELISA. Data are shown as mean ± SD.  ^∗∗^ *p* < 0.01 and  ^∗∗∗^ *p* < 0.001, compared with the control group. ^#^ *p* < 0.05, ^##^ *p* < 0.01, and ^###^ *p* < 0.001, compared with the CIA group.

To examine the in vivo anti‐inflammatory effects of triptolide, we established CIA mice model. The schematic protocol is shown in Figure 6A. Mice in the model group exhibited symmetrical joint erythema and stiffness compared to the controls (Figure 6B). After treatment with triptolide, arthritis symptoms in mice were significantly improved, as indicated by arthritis scores and hind paw thickness (Figure 6C,D). The microscopic changes of joint arthritis in CIA mice were assessed by H&E and Safranin O‐fast green staining. Our findings revealed the presence of inflammatory cell infiltration, synovial hyperplasia, and cartilage erosion in the model group; these pathologic changes were notably reversed after administration of triptolide (Figure 6E,F). Meanwhile, we evaluated the toxic side effects of triptolide in mice. As shown in Figure 7A, CIA modeling resulted in weight loss in mice, but there was no significant change in triptolide‐treated groups. Comprehensive serum biochemical analysis of hepatic function markers also revealed no significant differences between the triptolide‐treated and control groups, with all parameters remaining within normal ranges (Figure 7B,C). Furthermore, no obvious morphological changes or tissue damage were observed in the liver and kidney following triptolide treatment (Supporting information 3: Figure S4). These results indicate that triptolide at the administered dose exhibits a favorable safety profile.

### 3.11. Triptolide Exerts Antiarthritic Effects Through NR1D1‐Mediated STAT3 Signaling Inhibition in CIA Mice

To further elucidate the antiarthritic mechanism of triptolide, we performed western blots. With the establishment of the CIA model, the expression level of NR1D1 in ankle tissues decreased, followed by the activation of the STAT3 molecule. All treatment regimens—TP‐L and TP‐H—effectively inhibited STAT3 activation and restored NR1D1 expression (Figure 7D–F). In addition, we assessed the expression of inflammatory factors in the serum. Similar to the findings of our in vitro experiments, IL‐1β and IL‐6 levels were dramatically elevated in CIA mice, while triptolide dose‐dependently inhibited their release (Figure 7G,H). Together, these findings indicate that triptolide can mitigate arthritis symptoms in CIA mice by NR1D1‐mediated STAT3 signaling inhibition.

## 4. Discussion

Triptolide exhibits various pharmacological effects in RA, including anti‐inflammatory, immunosuppressive, and antiangiogenic effects [37]; however, its precise molecular mechanisms remain incompletely understood. To address this, our study integrated network pharmacology predictions, molecular docking simulations, and experimental validation to elucidate the core targets and regulatory mechanisms of triptolide in RA.

We first identified 100 potential targets of triptolide and performed an intersection analysis with 6786 potential RA–associated genes, yielding 73 candidate targets for triptolide intervention. Functional enrichment analysis revealed that these genes were significantly associated with transcription factor activation, regulation of inflammatory response, and cytokine/chemokine secretion—processes implicated in aberrant synovial fibroblast activation, vascular pannus formation, and progressive bone erosion in RA [38]. Module analysis of the PPI network identified STAT3 as a core gene, which plays a pivotal role in RA pathogenesis [39, 40]. To explore its upstream regulators, we reviewed the literature and identified NR1D1 as a candidate based on several lines of evidence. First, NR1D1 functions as a transcriptional repressor that directly antagonizes RORγt in Th17 cells and suppresses STAT3 transcription, positioning it upstream of the JAK/STAT pathway [41, 42]. Second, NR1D1 is a key circadian regulator involved in autophagy, immunity, and inflammation [43]. This is clinically relevant, as morning stiffness in RA patients exhibits marked diurnal variation, linking circadian disruption to disease progression. On the other hand, joint erosion is one of the hallmark features of RA. NR1D1 has been demonstrated to inhibit osteoclast differentiation, and its dysfunction may exacerbate osteoclast‐mediated bone resorption, leading to more severe joint erosion [28]. Given the known functions of NR1D1, targeting this molecule holds unique potential, particularly in coordinating circadian rhythms and inflammatory responses, offering valuable insights for RA treatment.

Synovial fibroblasts act as central mediators of RA pathogenesis, driving progressive joint destruction through their aberrant proliferation, invasive migration, and subsequent degradation of the cartilage matrix and bone [44–46]. RA synovitis is driven by a complex network of proinflammatory cytokines, and accordingly, multiple in vitro models have been established using different stimuli. Among these, TNF‐α is the most widely used and best‐characterized stimulus, capable of comprehensively recapitulating the typical pathological features of RA [47]. IL‐1β is commonly employed in studies focusing on cartilage destruction and bone erosion, while IL‐6 serves as an ideal tool for investigating the JAK‐STAT pathway and related targeted therapies [30, 48]. Building upon these established approaches, we stimulated synovial cells with 10 ng/mL TNF‐α to mimic the RA microenvironment and evaluate the anti‐inflammatory effects of triptolide. Our results demonstrated that triptolide dose‐dependently inhibited RA‐FLS proliferation, migration, invasion, and proinflammatory cytokine secretion, concomitant with the upregulation of NR1D1 and suppression of JAK1/STAT3 phosphorylation. Interestingly, TP‑L significantly inhibited JAK1 phosphorylation and IL‑1β/IL‑6 secretion but did not markedly affect NR1D1 expression or STAT3 phosphorylation. This discrepancy suggests that at lower concentrations, triptolide may suppress inflammation independent of the NR1D1/STAT3 axis, such as through the previously reported NF‐κB or MAPK pathways [37, 49]. To elucidate the underlying molecular mechanism, computational simulations (molecular docking and MD analysis) predicted that triptolide forms stable complexes with NR1D1 and STAT3. This prediction was further validated by two lines of experimental evidence. First, DARTS assays confirmed that triptolide directly binds to and stabilizes the NR1D1 protein, providing biochemical evidence for their physical interaction. Second, NR1D1 knockdown experiments demonstrated that depletion of NR1D1 largely abrogated the inhibitory effects of triptolide on JAK1/STAT3 phosphorylation and cell migration/invasion, indicating that NR1D1 is functionally required for triptolide‐mediated suppression of the JAK1/STAT3 pathway.

The CIA model, which recapitulates the core pathological features of human RA, provides a robust platform for evaluating novel therapies [50, 51]. In this model, triptolide treatment significantly ameliorated disease severity, as evidenced by reduced clinical scores and paw swelling, alongside decreased serum levels of IL‐6 and IL‐1β. These therapeutic effects were mechanistically linked to the upregulation of NR1D1 and concurrent suppression of STAT3 phosphorylation in joint tissues, corroborating our in vitro findings. Histopathological analysis further confirmed the alleviation of synovitis and cartilage erosion, consistent with prior reports [52, 53]. Importantly, at the administered dosage, triptolide did not induce significant body weight loss or hepatic toxicity, suggesting a favorable safety profile. Future efforts should focus on developing targeted delivery systems (e.g., nanoparticle‐based carriers) to enhance joint‐specific accumulation and minimize systemic exposure or exploring low‐dose triptolide in combination with conventional DMARDs to achieve synergy while mitigating toxicity [54, 55].

The current study has certain limitations. First, given that RA synovitis is driven by a complex cytokine network, models using other inflammatory mediators (e.g., IL‑1β, IL‑6, and IL‑17) should be incorporated to validate the broad applicability of triptolide. Second, although H&E and Safranin O‑fast green staining confirmed joint protection by triptolide, radiographic imaging such as micro‑CT remains necessary in future studies to provide three‑dimensional quantitative assessment of bone microarchitecture.

## 5. Conclusion

This study demonstrates that triptolide alleviates synovial inflammation and bone destruction in RA by activating NR1D1, which in turn inhibits the JAK1/STAT3 signaling pathway. Our findings elucidate a novel molecular mechanism underlying the antiarthritic effects of triptolide and provide a scientific rationale for further exploration of the NR1D1/JAK1/STAT3 axis as a potential therapeutic target for RA.

## Funding

This work was supported by the General Program of Zhejiang Provincial Administration of Traditional Chinese Medicine (Grant 2024ZR197), the Taizhou Science and Technology Program Project (Grant 24ywb88), and the Scientific Research Program of Affiliated Hospital of Zhejiang Chinese Medical University (Grant 2022FSYYZQ40).

## Ethics Statement

Animal experiments were approved by the Institutional Animal Care and Use Committee of Shanghai Rat & Mouse Biotechnology Co., Ltd. (Number LL‐2024‐112).

## Consent

The authors have nothing to report.

## Conflicts of Interest

The authors declare no conflicts of interest.

## Supporting Information

Additional supporting information can be found online in the Supporting Information section.

## Supporting information


**Supporting Information 1** Table S1: Causative genes of rheumatoid arthritis (RA).

**Supporting Information 2** Table S2: Potential targets of triptolide.

**Supporting Information 3** Figure S1: Protein–protein interaction (PPI) analysis of the potential targets and genes in the central module marked in red color. Figure S2: Effect of different concentrations of triptolide on viability of RA fibroblast‐like synoviocytes (RA‐FLS) assessed by CCK8. Figure S3: Efficiency of NR1D1 knockdown. RA‐FLS was transfected with NR1D1 siRNAs for 48 h and subjected to Western blot analysis. Figure S4: Comparison of morphological changes in livers and kidneys of mice in each group. Original magnification, ×200.

## Data Availability

The data that support the findings of this study are available from the corresponding author upon reasonable request.
