# Supplementary material for: NR1D1‐Mediated Suppression of JAK1/STAT3 Signaling Contributes to the Therapeutic Effects of Triptolide in Rheumatoid Arthritis
Source: Mediators Inflamm. 2026 Aug 25;2026:3971886. doi: 10.1155/mi/3971886 (PMC13504369; doi:10.1155/mi/3971886)
Supplement: Supplementary file 1 [file MI-2026-3971886-s003.docx]

Figure S1. Protein–protein interaction (PPI) analysis of the potential targets, genes in the central module marked in red color.

Figure S2. Effect of different concentrations of triptolide on viability of RA fibroblast-like synoviocytes (RA-FLS) assessed by CCK8.

Figure S3. Efficiency of NR1D1 knockdown. RA-FLS was transfected with NR1D1 siRNAs for 48 h and subjected to Western blot analysis.

Figure S4 Comparison of morphological changes in livers and kidneys of mice in each group. Original magnification, ×200.
